# Influence of drying temperature and storage period on the quality of cherry and plum tomato powder

**DOI:** 10.1002/fsn3.658

**Published:** 2018-04-25

**Authors:** Adewale Obadina, Jumoke Ibrahim, Ifeoluwa Adekoya

**Affiliations:** ^1^ Department of Food Science and Technology Federal University of Agriculture Abeokuta Ogun State Nigeria; ^2^ Department of Biotechnology and Food Technology University of Johannesburg Johannesburg South Africa

**Keywords:** ascorbic acid, color, drying, lycopene, tomato

## Abstract

The quality changes of cherry and plum tomatoes dried at different temperatures (60, 65 and 70°C) milled into powder and stored for 8 weeks were assessed in this study. The ascorbic acid and lycopene content of the tomato powders were significantly different with values that ranged from 5.10 to 7.70 mg/100 g and 211.53 to 246.02 mg/kg, respectively. Color parameters redness (a*) and chroma decreased, while lightness (L*), yellowness (b*), and hue increased as the drying temperature increased. In addition, increase was observed in the total fungal load and lightness of the two tomato varieties at all temperatures, while the titratable acidity, pH, ascorbic acid, lycopene, redness, and yellowness increased as the storage period increased to 8 weeks. The 60°C dried plum tomato powder had the best result in terms of quality retention at the end of the storage period. Some quality parameters increased and decreased with drying and storage of plum and cherry tomato powders.

## 1 INTRODUCTION

Globally, tomatoes (*Lycopersicum esculentum*) have become one of the most widely grown and consumed vegetable and they come in various sizes, shapes, colors, and varieties. They are excellent sources of ascorbic acid, anthocyanins, phenolic compounds, and carotenoids particularly lycopene, all which have high antioxidant activities (Shi & Le Maguer, 2000). Tomato has the ability to improve the quality, acceptance, and satiety value of diets and can be consumed both as raw and processed products. The cherry and plum tomatoes are the most widely grown varieties in Nigeria among hundreds of tomato varieties grown worldwide, the latter are usually oval in shape with more solid content compared with the former, which are generally round shaped; plum tomatoes are usually considered to be more suitable for processing into sauces and pastes. With the benefit of tomatoes also lies its shortcomings, they are highly perishable in the fresh state due to their high moisture content leading to wastages and losses during harvesting and storage especially in sub‐Saharan Africa. Losses in tomato productions are also accrued to poor postharvest handling practices. Therefore, the prevention of these losses and wastage is paramount especially in countries like Nigeria whose populace are all year round heavy tomato consumers and there is subsequent imbalance in demand and supply at the harvesting off‐seasons (Akanbi, Adeyemi, & Ojo, 2006). These shortcomings can, however, be reduced through the processing and conversion of tomato into other forms such as tomato powder as well as the investigation of technologies that can reduce packaging, handling, and transportation cost together with the increased shelf life. In view of this, it also important to emphasize the rapidly increasing demand of dehydrated tomato in domestic and in international markets with major portion of it being used for preparation of convenient foods (Davoodi, Vijayanand, Kulkarni, & Ramana, 2007). In order to drive in possible solutions to these problems, there is need to understand the impact of different processing techniques on the quality of tomato products, such as tomato powder. Some of the important quality parameters of tomatoes are appearance (color, taste, size, and shape), firmness, flavor, nutritional value, and microbial safety. However, a significant number of studies have addressed the effect of some processing techniques and conditions on the quality of tomatoes (Kim & Chin, 2016; Liu, Cao, Wang, & Liao, 2010; Tigist, Workneh, & Woldetsadik, 2013). Das Purkayastha, Nath, Deka, and Mahanta (2013) assessed the effect of drying temperatures on tomato slices, while Correia, Loro, Zanatta, Spoto, and Veira (2015) evaluated the effect temperature, time, and material thickness on the dehydration process of tomato. To the best of our knowledge, only few studies have focused on the impact of drying conditions and storage periods on the quality of tomato powder from the two varieties of tomato understudied. This research was therefore aimed at assessing the effect of different drying and storage conditions on the quality and shelf stability of powdered cherry and plum tomatoes.

## 2 MATERIALS

Two varieties of wholesome and fresh ripe tomato fruits (plum and cherry) were purchased from two markets (Ita‐oshin and Osiele) in Ogun state, Nigeria in July 2016.

### 2.1 Sample preparation

The tomatoes were visually sorted based on color (bright red), firmness, size, and absence of physical damage. After which they were washed with sterile, distilled water to remove dirts and soils, cut into slices with thickness of 10 mm, drained, placed in an air oven (LCO‐3050H; Labtech, Limited, Korea) at temperatures of 60, 65 and 70°C, respectively for 18 hr using the thin layer procedure as described by Akanbi et al.(2006). The slices were then milled into fine powder with the aid of a blender, allowed to cool, sieved through a 500 μm aperture size sieve and packaged in polyethylene films and were subjected to the following analysis.

## 3 METHODOLOGY

### 3.1 Moisture content determination

The moisture content of the tomato powders was determined according to the method 931.04 of AOAC (2005).

### 3.2 Determination of ascorbic acid

Ascorbic acid content of the tomato powder was determined using the 2,6‐dichlorophenolindophenol AOAC method (1990). Briefly, 3% of 50 ml metaphosphoric acid was added to 10 g of tomato powder in a volumetric flask, after which it was centrifuged in a centrifuge (Hettich Zentrifugen Company, Germany) at 4,000 g for 15 min and titrated to a pink endpoint with a standard dye. The ascorbic acid content (%) was calculated from the dye factor, titration value, volume, and dilution of the sample.

### 3.3 Determination of lycopene content

Tomato powder (1.0 g) of each sample was accurately weighed into 125 ml Erlenmeyer flask, then 100 ml of hexane:ethanol:acetone in ratio 2:1:1 was added. The flask was then sealed with a rubber stopper, after 30 min of extraction, the absorbance of the supernatant containing lycopene was measured at 503 nm using a spectrophotometer (Buck Model 20A; Buck Scientific, East Norwalk, CT, USA).

$$\text{Lycopene}\;\text{(mg/kg)}=(A_{503}\times 171.7)/W$$

where

A_503_ = absorbance at 503 nm

W = weight of sample

### 3.4 Determination of pH

The pH of the tomato powder was determined using a pH meter (Jenway, Model 3510; Essex, UK) after calibration using standard buffers of pH 4.00 and 7.00.

### 3.5 Determination of titratable acidity (TA)

Titratable acidity was determined using the AOAC (2005) method: the amount of acid (citric acid) in each sample was determined by using the following equation:

%Titratableacidity=Titre×0.1×0.00705×100/Weightofsample

### 3.6 Color analysis

Color measurements of the tomato powder were done using a colorimeter (CR‐400; Konica Minolta, Tokyo, Japan). Before measuring the color, a standard white ceramic plate was used to calibrate the colorimeter (L* = 96; a* = 0.13; b* = 1.63). L* denotes lightness (L* = 0 for black, L* = 100 for white), a* denotes intensity in red‐green (a* > 0 for red, a* < 0 for green), b* denotes intensity in blue to yellow (b* > 0 for yellow, b* < 0 for blue). Other color parameters that were calculated are:

$$\text{Redness}\;\text{to}\;\text{yellowness}\;\text{ratio}\left(a^{*}/b^{*}\right)=a^{*}/b^{*}$$

$$\text{Chroma}\left(C^{*}\right)=\sqrt{{\left(a^{2}+b\right)}^{2}}$$

$$\text{Hue}\left(h\right)=[tan-1\left(b^{*}/a^{*}\right)]$$

### 3.7 Microbial population

One gram of each tomato powder sample was homogenized at 5,000 g using a homogenizer (ultra‐turrax T25 blade‐type homogenizer; Ika‐Werk, Staufen, Germany) in 9.0 ml sterile 0.1% peptone water for 30 s and then a tenfold serial dilution was carried out. Aliquot of 1 ml was plated Nutrient agar (CM003; Oxoid Ltd, Basingstoke Hants, England) and Malt Extract Agar (CM 0099; Oxoid) being suitable media for the growth of total aerobic bacteria and fungi, respectively. Plated aliquots for total aerobic bacteria were incubated at 37°C for 24 hr, while fungi plated aliquots were incubated at 25°C for 5 days. A colony counter (LM‐10; Analab, New Delhi, India) was used to enumerate the microbial population and the counts of the microorganisms were recorded in cfu/g.

### 3.8 Storage studies

Polyethylene‐packaged cherry and plum 60, 65 and 70°C dried tomato powders stored at ambient temperature (25 ± 2°C) for 8 weeks were analyzed at 2‐week interval for a period of 8 weeks to determine the changes in their quality attributes: pH, TA, microbial load, color parameters, ascorbic acid, lycopene, and moisture contents. The percentage increase/decrease in these quality attributes the end of the storage period was calculated as;

$$\begin{array}{cc} & \text{Quality}\;\text{parameter}\;\text{at}\;\text{week}\;0/8-\text{quality}\;\\ & \text{parameter}\;\text{at}\;\text{week}\;8/0\times 100 \end{array}$$

Also, change in color at the end of the storage period was calculated by the following equation:

$$\Delta E^{*}={\left[{\left(L_{0}^{*}-L^{*}\right)}^{2}+{\left(a_{0}^{*}-a^{*}\right)}^{2}+{\left(b_{0}^{*}-b^{*}\right)}^{2}\right]}^{1/2}$$

where L_0_* = Lightness value of cherry/plum tomato powder at week 0

L* = Lightness of cherry/plum tomato powder at 8th week of storage

a_0_* = Redness value of cherry/plum tomato powder at week 0

a* = Redness of cherry/plum tomato powder at 8th week of storage

b_0_* = Yellowness value of cherry/plum tomato powder at week 0

b* = Yellowness of cherry/plum tomato powder at 8th day of storage

### 3.9 Statistical analysis

All experiments were performed in triplicates, and data collected were subjected to ANOVA (analysis of variance). Duncan Multiple Range test was used to separate the differences among the means, and significances were accepted at 5% confidence level (Duncan, 1955) using SPSS version 23 for windows.

## 4 RESULTS AND DISCUSSION

### 4.1 Chemical properties of cherry and plum tomato powder dried at different temperatures

The chemical composition of tomato powder from two tomato varieties dried at different temperatures is presented in Table 1. The moisture, vitamin, lycopene, and TA of the different varieties tomato powder were significantly different with values that ranged from 11.25% to 13.75%, 5.10 to 0.77 mg/100 g, 211.53 to 246.02 mg/kg, and 0.71% to 1.10%, respectively. Plum tomato dried at 60°C had the highest value for moisture and ascorbic acid content, while plum tomato dried at 70°C had the lowest value, and this can be due to the higher drying temperature of the aforementioned plum tomato. Cherry tomato dried at 70°C had the lowest value for lycopene, which is the most abundant carotenoid (Periago et al., 2009), and the deep‐red coloring pigment was found in tomatoes and its products (Shi & Le Maguer, 2000). They also exhibit antioxidant properties thereby contributing to the elimination of free radicals in the body. The lycopene content of the analyzed tomato powders ranged from 211.53 to 246.02 mg/kg; lycopene content of tomato paste was between 104.78 and 923.45 mg/kg in the study of Eke‐Ejiofor (2015). Asides for genetic variability, processing at higher temperature might have facilitated the higher lycopene content in the case of cherry and plum tomato powders dried at 65°C. Nguyen and Schwartz (1998) reported that the availability of lycopene from tomato products is increased when they are processed at high temperature or packaged with oil. The authors also reported that mechanical treatment (homogenization) and heating enhances the release of lycopene from the tomato matrix.

**Table 1:** Chemical properties of cherry and plum tomato powders at different drying temperatures

Samples	pH	Moisture (%)	Vitamin C (mg/100 g)	Lycopene (mg/kg)	TA (%)
Cherry tomato dried at 60°C	4.28 ± 0.05^a^	13.55 ± 0.14^c^	7.50 ± 0.01^c^	227.13 ± 7.07^ab^	0.71 ± 0.01^a^
Cherry tomato dried at 65°C	4.29 ± 0.04^a^	13.75 ± 0.01^c^	7.20 ± 0.21^c^	242.17 ± 4.24^c^	0.83 ± 0.01^b^
Cherry tomato dried at 70°C	4.22 ± 0.04^a^	13.20 ± 0.14^c^	7.20 ± 0.00^c^	211.53 ± 2.12^a^	0.99 ± 0.01^c^
Plum tomato dried at 60°C	4.22 ± 0.03^a^	13.20 ± 0.01^d^	7.70 ± 0.10^c^	214.59 ± 14.84^ab^	0.74 ± 0.01^a^
Plum tomato dried at 65°C	4.19 ± 0.04^a^	12.85 ± 0.35^b^	6.00 ± 0.13^b^	246.02 ± 8.48^c^	0.99 ± 0.01^c^
Plum tomato dried at 70°C	4.24 ± 0.03^a^	11.25 ± 0.14^a^	5.10 ± 0.01^a^	233.19 ± 1.41^bc^	1.10 ± 0.14^c^

Values represent mean and standard deviation. Data with the same superscript across the column are not significantly (*p* ≤ .05) different. TA, titratable acidity

Nutritionally, a food manufacturer would make effort to minimize the loss of vitamins during processing and storage. Therefore, the concentration of ascorbic acid in tomato powder can be counted upon as an indicator of processing conditions as well as raw material quality. Tomato is a good source of ascorbic acid and it is of great important to investigate the level of residual ascorbic acid in tomato powder. As the drying temperature increased, the ascorbic acid content of plum tomato reduced significantly. Heat and oxygen are among the main factors causing loss of ascorbic acid in tomato processing (Soto‐Zamora, Yahia, Brecht, & Gardea, 2005). Ascorbic acid is heat labile, and about 30% of ascorbic acid is degraded during drying and the drying temperature may have influenced the ascorbic acid. Oxygen also reacts with ascorbic acid leading to oxidation, wherein ascorbic acid is broken down to dehydroascorbic acid, followed by hydrolysis into 2,3‐diketogulonic acid and further oxidation and polymerization to a number of nutritionally inactive products (Cernisev, 2010). This reaction is also accelerated under high‐temperature conditions. An insignificant reduction in ascorbic was observed in the case of the cherry tomatoes at increasing temperature compared to plum tomato, which might be due to species variation in terms of morphology and moisture content. The rate of degradation of the ascorbic acid content of cherry tomatoes will also depend largely on its initial ascorbic acid content, which was not accounted for in this study but it was clear that the cherry tomatoes had higher ascorbic acid content than plum tomatoes.

The pH of the tomato powder from different tomato varieties ranged from 4.19 to 4.29, and the processing conditions had no significant effect on the pH. Tomato products are generally grouped as acidic foods (pH <4.6), and the pH range obtained from this study were in line with this. Microbial contamination of tomatoes consequently leads to deterioration and represents a health hazard to the consumer, however, mesophylls, molds, yeast, and coliforms are the commonly found microorganisms in tomato (Vinha, Barreira, Castro, Costa, & Oliveira, 2013). The total aerobic and total fungal counts of the tomato powder samples ranged from 5.15 × 10^2^ to 5.56 × 10^2^ cfu/g and 7.30 × 10^1^ to 7.55 × 10^1^ cfu/g, respectively (Table 2). The levels of contamination observed may be derived from direct contact with handlers, air, and storage environment among others.

**Table 2:** Microbial qualities of cherry and plum tomato powders at different drying temperatures

Samples	Total fungal count (×10^1^ cfu/g)	Total aerobic count (×10^2^ cfu/g)
Cherry tomato dried at 60°C	7.50 ± 0.14^a^	5.56 ± 0.07^b^
Cherry tomato dried at 65°C	7.40 ± 0.14^a^	5.40 ± 0.14^ab^
Cherry tomato dried at 70°C	7.30 ± 0.21^a^	5.15 ± 0.21^a^
Plum tomato dried at 60°C	7.55 ± 0.07^a^	5.55 ± 0.07^b^
Plum tomato dried at 65°C	7.45 ± 0.14^a^	5.45 ± 0.07^ab^
Plum tomato dried at 70°C	7.40 ± 0.21^a^	5.40 ± 0.14^ab^

Values represent mean and standard deviation. Data with the same superscript across the column are not significantly (*p* ≤ .05) different.

The a*/b* ratio of the tomato powder from different tomato varieties dried at different temperature was significantly (*p* ≤ .05) different with values that ranged from 0.91 to 1.26 (Table 3). As the temperature increased, the color intensity parameters (a*, b*) decreased for the two tomato varieties. Hayes, Smith, Morris, and Smith (1998) reported a*/b* ratio as the indicative and quality parameter for the color of tomato products, while Sobowale, Olatidoye, Odunmbaku, and Raji (2012) stated that an a*/b* ratio of 1.9 or greater represents a quality tomato paste in terms of color. The a*/b* ratio obtained in this study was lower than 1.9 which indicates that the temperature negatively affected the color of the tomato powder. The values of chroma followed the sequence of cherry tomato dried at 70°C, cherry tomato dried at 60°C, plum tomato dried at 70°C, cherry tomato dried at 65°C, plum tomato dried at 65°C, and plum tomato dried at 60°C (Table 3); this confirms that there was more retention of the redness color of the tomato at lower temperatures.

**Table 3:** Color intensity of cherry and plum tomato powders at different drying temperatures

Samples	L*	a*	b*	a*/b*	Chroma	Hue
Cherry tomato dried at 60°C	32.09 ± 1.29^ab^	17.59 ± 0.26^b^	15.18 ± 1.48^a^	1.15 ± 0.07^c^	324.59 ± 6.75^b^	0.71 ± 0.05^bc^
Cherry tomato dried at 65°C	34.99 ± 2.44^bc^	17.71 ± 0.92^b^	17.86 ± 1.28^b^	0.99 ± 0.02^b^	331.50 ± 7.34^b^	0.79 ± 0.02^c^
Cherry tomato dried at 70°C	36.22 ± 0.51^c^	16.01 ± 0.79^a^	17.75 ± 0.68^b^	0.91 ± 0.01^a^	274.01 ± 7.29^a^	0.84 ± 0.04^c^
Plum tomato dried at 60°C	30.65 ± 0.18^a^	19.75 ± 0.46^c^	14.07 ± 0.36^a^	1.21 ± 0.13^c^	404.12 ± 6.28^c^	0.62 ± 0.04^a^
Plum tomato dried at 65°C	31.67 ± 0.73^a^	19.22 ± 3.13^c^	15.21 ± 0.11^a^	1.26 ± 0.15^c^	384.62 ± 9.22^c^	0.67 ± 0.01^a^
Plum tomato dried at 70°C	31.54 ± 0.48^a^	17.76 ± 1.37^b^	14.53 ± 0.72^a^	1.22 ± 0.13^c^	329.95 ± 8.79^b^	0.67 ± 0.02^a^

Values represent mean and standard deviation. Data with the same superscript across the column are not significantly (*p* ≤ .05) different.

### 4.2 Chemical properties of cherry and plum tomato powder stored for 8 weeks

**Figure 1:**
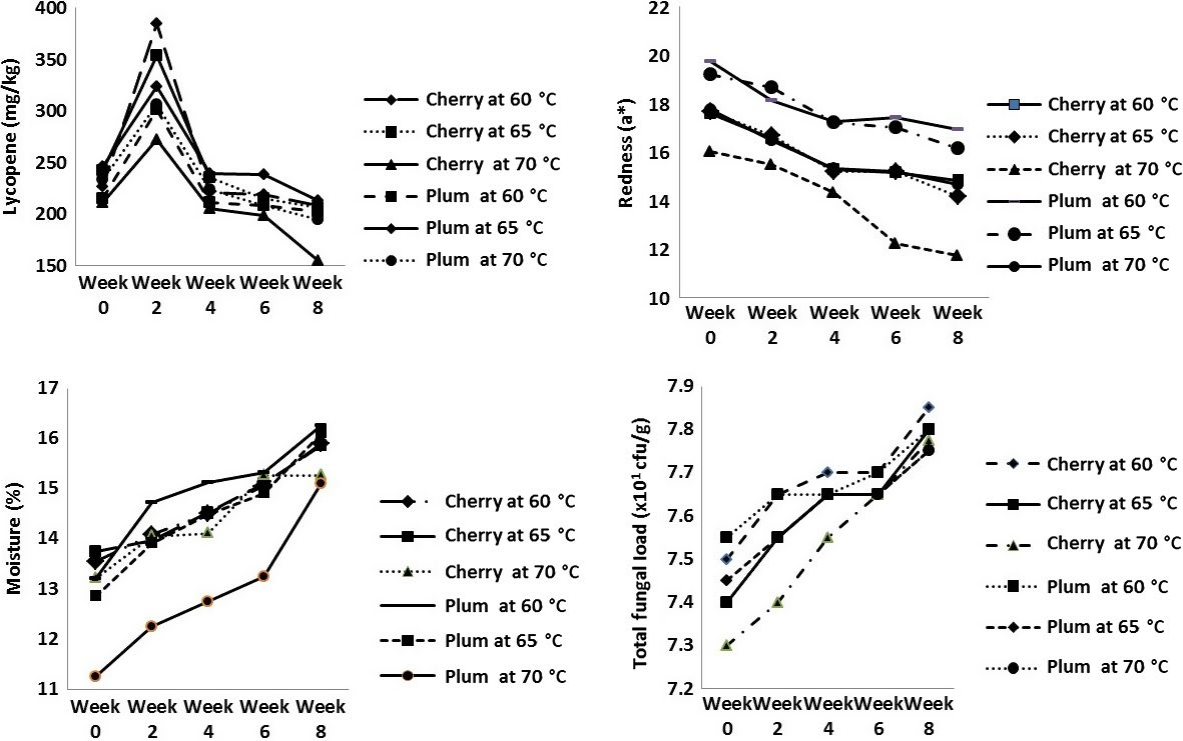
Lycopene, redness, moisture content, and total fungal load of cherry and plum tomatoes during storage

Low‐temperature storage has been identified as the best method of maintaining the quality of most vegetables and fruits including tomatoes due to its impact on ripening, transpiration, and reduced respiration rate among others (Tigist et al., 2013). In dry and powdery states of fruits and vegetables, compared to high‐temperature storage, low‐temperature storage is also beneficial due to its cryoprotectant effects against nutrients such as vitamins (Cernisev, 2010) and reduced degradative reactions as a result of decreased water mobility inside powder particles (Rodriguez et al., 2009). Nevertheless, under the condition of this study, the tomatoes were stored for a period of 8 weeks under ambient temperature based on the conventional temperature in tomato‐producing areas of the country and the perception into the preferred storage method of tomato powder by consumers as this could have a better implication for knowing the quality of the powdered tomatoes. There were variations in the chemical properties of the cherry and plum tomato powder stored for 8 weeks as shown in Figure 1, Tables 4 and 5. Increase was observed in moisture content, total fungal load, total aerobic bacteria load, and the lightness of the samples, while the TA, pH, ascorbic acid, lycopene, redness, and yellowness decreased as the storage period increased. The increase in moisture content could be because of the conditions of the storage environment such as temperature and relative humidity as well as the physiological activities of the tomato powder. pH and TA are both acidity indicators in tomatoes and the decrease observed in the TA during storage could be due to breakdown of organic acids during storage, similar results were reported by Castro, Vigneault, Therese, and Cortez (2005). In addition to this, the significant differences in the pH observed as percentage increase in Table 4 could be due to genotypic variability (Mohammed, Wilson, & Gomes, 1999) and were consistent with the finding of Tigist et al. (2013) although they worked on fresh tomato.

**Table 4:** Percentage increase in the quality parameters of cherry and plum tomato powders at the end of 8 weeks storage period

Sample	pH	Moisture content	Total fungal count	Total aerobic count	Lightness
Cherry tomato dried at 60°C	1.91 ± 0.25^b^	17.34 ± 0.07^c^	4.67 ± 0.14^b^	3.60 ± 0.14^a^	0.90 ± 0.13^a^
Cherry tomato dried at 65°C	2.85 ± 0.11^c^	15.27 ± 0.35^ab^	5.40 ± 0.21^c^	5.48 ± 0.29^c^	1.03 ± 0.11^a^
Cherry tomato dried at 70°C	1.16 ± 0.09^a^	15.53 ± 0.28^b^	5.50 ± 0.07^c^	5.50 ± 0.07^c^	1.05 ± 0.02^a^
Plum tomato dried at 60°C	3.45 ± 0.65^c^	13.10 ± 0.28^a^	3.31 ± 0.14^a^	4.72 ± 0.19^b^	1.13 ± 0.04^a^
Plum tomato dried at 65°C	2.81 ± 0.05^c^	15.29 ± 0.21^ab^	4.22 ± 0.35^b^	3.31 ± 0.14^a^	1.42 ± 0.13^a^
Plum tomato dried at 70°C	1.85 ± 0.31^ab^	14.22 ± 0.35^a^	4.72 ± 0.14^b^	4.02 ± 0.35^ab^	2.20 ± 0.15^b^

Values represent mean and standard deviation. Data with the same superscript across the column are not significantly (*p* ≤ .05) different.

**Table 5:** Percentage decrease in the quality parameters of cherry and plum tomato powders at the end of 8 weeks storage period

Sample	Vitamin C (mg/100 g)	Lycopene (mg/kg)	Redness	Yellowness	TA (%)
Cherry tomato dried at 60°C	13.33 ± 1.38^a^	8.27 ± 1.16^b^	15.50 ± 0.49^a^	14.54 ± 0.24^a^	18.42 ± 2.12^c^
Cherry tomato dried at 65°C	25.21 ± 2.34b^b^	15.18 ± 1.36^c^	19.89 ± 0.48^b^	17.42 ± 0.98^b^	24.28 ± 2.12^d^
Cherry tomato dried at 70°C	27.91 ± 3.94^b^	26.75 ± 1.09^d^	20.74 ± 0.71^b^	19.19 ± 0.23^bc^	27.84 ± 1.26^de^
Plum tomato dried at 60°C	13.33 ± 3.13^a^	5.62 ± 0.52^a^	14.20 ± 0.99^a^	14.29 ± 1.02^a^	8.33 ± 2.12^a^
Plum tomato dried at 65°C	25.23 ± 4.84^b^	13.51 ± 1.14^c^	15.97 ± 0.82^a^	16.61 ± 1.09^a^	13.63 ± 1.79^b^
Plum tomato dried at 70°C	28.38 ± 3.76^b^	16.67 ± 1.31b^c^	17.46 ± 0.41^ab^	17.49 ± 0.36^a^	18.06 ± 2.36^c^

Values represent mean and standard deviation. Data with the same superscript across the column are not significantly (*p* ≤ .05) different. TA, titratable acidity

Changes in ascorbic acid are usually used to obtain the overall nutrient retention of food products; in this study, ascorbic acid degraded as the storage period increased and the decrease was more prominent in cherry and plum tomato dried at 70°C with both having 27.91% and 28.38% decrease, respectively. This might be due to the high sensitivity of ascorbic acid to oxidation during storage and its easy depletion due to the application of heat (Davey et al., 2000; Murcia, Lopez‐Ayerra, Martinez‐Tome, Vera, & Garcia‐Carmona, 2000) and this might facilitate ascorbic acid supplementation in products such as tomato powder.

The result of the microbiological parameters during storage reveals the existence of a correlation with physiological parameters of the tomatoes understudied particularly the pH and moisture content which increased during storage by a maximum of 2.85% and 17.34%, respectively in cherry tomato. In the study of Vinha et al. (2013) on the influence of storage conditions on the qualities of three different tomato cultivars, the microbial load of the cultivars increased as the storage period increased except for the *cereja* cultivar.

Color is a critical factor of the consumer preference for any tomato product including powder, and a* (redness) is the most important parameter for measuring tomato color. When red color pigments started to decrease, an increase in L* value indicated the lightening of the red color. In general, higher values of coordinate L* were noted for cherry tomato powder regardless of the temperature used when compared to plum tomato powder which might be due to the differences in the color pigment of the two tomato varieties. An influence of the drying temperatures in case of color attribute b* was observed in cherry tomato powder pointing to the probable presence of newly formed compounds (Pathare, Opara, & Al‐Said, 2013).

**Figure 2:**
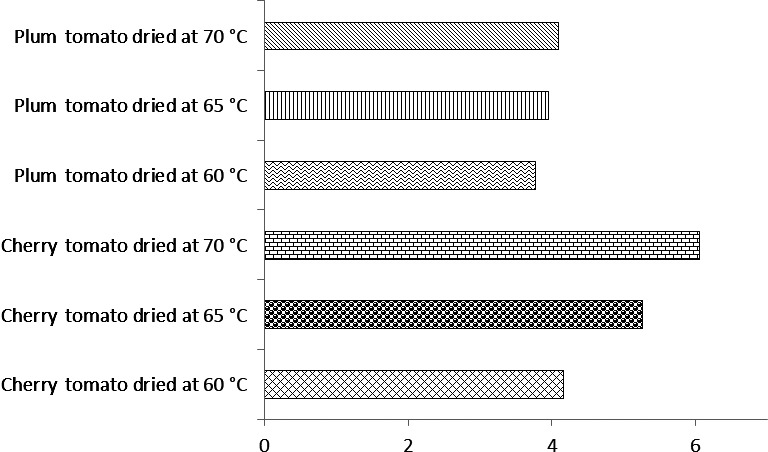
Change in color of cherry and plum tomato powders at the end of 8 weeks storage period

Change in color gradually increased throughout the storage periods in all samples (Figure 2). ΔE indicates the change of all color parameters and is more attuned than the b*, a*, L* value. ΔE increased as the storage period increased with cherry tomato powder having higher values than plum tomato powder. According to Urbanyi and Horti (1989), the typical color change of stored tomato powders is usually characterized as increase in yellowness, decrease in redness, and concurrent lightening of the color. The gradual decrease in redness and the observed color change during days of storage could be associated with the breakdown of lycopene a major contributor to tomato color due to its bright red carotene and carotenoid pigment. Plum tomato dried at 70°C had the highest lycopene content (385 mg/kg) at week 2 but decreased at the end of the 8th week. Due to the existence of a long chain of conjugated carbon‐carbon double bonds, lycopene is susceptible to chemical changes upon exposure to light and heat (D'Souza, Singhq, & Ingle, 1992). Undesirable degradation is unavoidable due to oxidation and isomerization of translycopene to the *cis* isomers during processing which facilitate reduction in the biological activity of lycopene as well as color loss (Edwards & Lee, 1986).

## 5 CONCLUSION

The study showed that there were significant (*p* ≤ .05) differences in chemical properties between the tomato varieties during drying. Cherry tomato powder had higher value for lightness, ascorbic acid, hue, yellowness, and moisture than plum tomato powder, whereas plum tomato powder had higher values for TA, chroma, and redness. Drying of cherry tomatoes at 60 to 70°C had no significant effect on its pH, moisture, ascorbic acid content, total fungal load, and lightness on the other hand; drying of plum tomatoes within the stated temperature range had significant effect on these parameters except for lightness and pH. Increase was observed in the total fungal load and lightness of the two tomato varieties at all temperatures, while the TA, pH, ascorbic acid, lycopene, redness, and yellowness increased as the storage period increased to 8 weeks. In terms of ascorbic acid, redness, moisture content, lycopene, yellowness, and TA at the end of the 8 weeks storage period, 60°C plum tomato powder had the minimum effect. This study will be useful in establishing appropriate storage and processing conditions to minimize degradation of quality parameters of tomato powder and recommends possible supplementation of ascorbic acid in tomato powder during drying as well as the reduction in tomato processing temperatures.

## CONFLICT OF INTEREST

The authors have no conflict of interest to declare.

## References

[fsn3658-bib-0001] Akanbi, C. T. , Adeyemi, R. S. , & Ojo, A. (2006). Drying characteristics and sorption isotherm of tomato slices. Journal of Food Engineering, 73(2), 157–163. https://doi.org/10.1016/j.jfoodeng.2005.01.015

[fsn3658-bib-0002] AOAC (1990). Official methods of analysis of AOAC International In Association of Official Analysis Chemists International (Vol. II, pp. 1058–1059).

[fsn3658-bib-0003] AOAC (2005). Official methods of analysis (19th ed.). Washington, DC: Association of Analytical Chemists, USA.

[fsn3658-bib-0004] Castro, L. R. , Vigneault, C. , Therese, C. M. , & Cortez, L. A. B. (2005). Effect of cooling delay and cold‐chain breakage on “Santa Clara” tomato. Journal of Food, Agriculture and Environment, 3(1), 49–54.

[fsn3658-bib-0005] Cernisev, S. (2010). Effects of conventional and multistage drying processing on non‐enzymatic browning in tomato. Journal of Food Engineering, 96(1), 114–118. https://doi.org/10.1016/j.jfoodeng.2009.07.002

[fsn3658-bib-0006] Correia, A. F. K. , Loro, A. C. , Zanatta, S. , Spoto, M. H. F. , & Veira, T. M. S. F. (2015). Effect of temperature, time, and material thickness on the dehydration process of tomato. International Journal of Food Science, 2015, 970724 https://doi.org/10.1155/2015/970724 26904666 10.1155/2015/970724 PMC4745559

[fsn3658-bib-0007] Das Purkayastha, M. , Nath, A. , Deka, B. C. , & Mahanta, C. L. (2013). Thin layer drying of tomato slices. Journal of Food Science and Technology, 50(4), 642–653. https://doi.org/10.1007/s13197-011-0397-x 24425966 10.1007/s13197-011-0397-x PMC3671039

[fsn3658-bib-0008] Davey, M. W. , Van Montagu, M. , Inze, D. , Sanmartin, M. , Kanellis, A. , Smirnoff, N. , & Fletcher, J. (2000). Plant L‐ascorbic acid: Chemistry, function, metabolism, bioavailability and effects of processing. Journal of the Science of Food and Agriculture, 80(7), 825–860. https://doi.org/10.1002/(ISSN)1097-0010

[fsn3658-bib-0009] Davoodi, M. G. , Vijayanand, P. , Kulkarni, S. G. , & Ramana, K. V. R. (2007). Effect of different pre‐treatments and dehydration methods on quality characteristics and storage stability of tomato powder. LWT – Food Science and Technology, 40(10), 1832–1840. https://doi.org/10.1016/j.lwt.2006.12.004

[fsn3658-bib-0010] D'Souza, M. C. , Singhq, S. , & Ingle, M. (1992). Lycopene concentration of tomato fruit can be estimated from chromaticity values. Horticulture Science, 27, 465–466.

[fsn3658-bib-0011] Duncan, D. (1955). Multiple range and multiple F tests. Biometrics, 11(1), 1–42. https://doi.org/10.2307/3001478

[fsn3658-bib-0012] Edwards, C. G. , & Lee, C. Y. (1986). Measurement of provitamin A carotenoids in fresh and canned carrots and green peas. Journal of Food Science, 51(2), 534–535. https://doi.org/10.1111/j.1365-2621.1986.tb11180.x

[fsn3658-bib-0013] Eke‐Ejiofor, J. E. (2015). Comparative evaluation of lycopene content and some chemical properties of commonly consumed brands of tomato paste in Port – Harcourt, South‐South, Nigeria. Journal of Food and Nutrition Sciences, 3(2), 35.

[fsn3658-bib-0014] Hayes, W. A. , Smith, F. G. , Morris, A. E. J. , & Smith, P. G. (1998). The production and quality of tomato concentrates. Critical Reviews in Food Science and Nutrition, 38(7), 537–564. https://doi.org/10.1080/10408699891274309 9813734

[fsn3658-bib-0015] Kim, H. S. , & Chin, K. B. (2016). Effects of drying temperature on antioxidant activities of tomato powder and storage stability of pork patties. Korean Journal for Food Science of Animal Resources, 36(1), 51–60. https://doi.org/10.5851/kosfa.2016.36.1.51 27499664 10.5851/kosfa.2016.36.1.51 PMC4973938

[fsn3658-bib-0016] Liu, F. , Cao, X. , Wang, H. , & Liao, X. (2010). Changes of tomato powder qualities during storage. Powder Technology, 204(1), 159–166. https://doi.org/10.1016/j.powtec.2010.08.002

[fsn3658-bib-0017] Mohammed, M. , Wilson, L. A. , & Gomes, P. I. (1999). Postharvest sensory and physiochemical attributes of processing and non‐processing tomato cultivars. Journal of Food Quality, 22(2), 167–182. https://doi.org/10.1111/j.1745-4557.1999.tb00549.x

[fsn3658-bib-0018] Murcia, M. A. , Lopez‐Ayerra, B. , Martinez‐Tome, M. , Vera, A. M. , & Garcia‐Carmona, F. (2000). Evolution of ascorbic acid and peroxidase during industrial processing of broccoli. Journal of the Science of Food and Agriculture, 80(13), 1882–1886. https://doi.org/10.1002/(ISSN)1097-0010

[fsn3658-bib-0019] Nguyen, M. L. , & Schwartz, S. J. (1998). Lycopene stability during food processing. Proceedings of the Society for Experimental Biology and Medicine, 218(2), 101–105. https://doi.org/10.3181/00379727-218-44274 9605205 10.3181/00379727-218-44274

[fsn3658-bib-0020] Pathare, P. B. , Opara, U. L. , & Al‐Said, F. A. (2013). Colour measurement and analysis in fresh and processed foods: A review. Food Bioprocess Technology, 6, 36–60. https://doi.org/10.1007/s11947-012-0867-9

[fsn3658-bib-0021] Periago, M. J. , García‐Alonso, J. , Jacob, K. , Belén Olivares, A. , José Bernal, M. , Dolores Iniesta, M. , & Ros, G. (2009). Bioactive compounds, folates and antioxidant properties of tomatoes (*Lycopersicum esculentum*) during vine ripening. International Journal of Food Sciences and Nutrition, 60(8), 694–708. https://doi.org/10.3109/09637480701833457 19919517 10.3109/09637480701833457

[fsn3658-bib-0022] Rodriguez, H. , Peixoto, K. , Jaeger, L. , Rodriguez, L. , Pedrosa, C. , Trinidade, A. , & Pierucci, R. (2009). Legumes seeds protein isolates in the production of ascorbic acid microparticles. Journal of Food Research International, 42(1), 115–121.

[fsn3658-bib-0023] Shi, J. , & Le Maguer, M. (2000). Lycopene in tomatoes: Chemical and physical properties affected by food processing. Critical Reviews in Biotechnology, 20, 293–334. https://doi.org/10.1080/07388550091144212 11192026 10.1080/07388550091144212

[fsn3658-bib-0024] Sobowale, S. S. , Olatidoye, O. P. , Odunmbaku, L. A. , & Raji, O. H. (2012). A comparative study on physicochemical and rheological properties of imported tomato paste in Nigeria. Sustainable Agriculture Research, 1(2), 51–56.

[fsn3658-bib-0025] Soto‐Zamora, G. , Yahia, E. M. , Brecht, J. K. , & Gardea, A. (2005). Effects of postharvest hot‐air treatments on the quality and antioxidant levels in tomato fruit. LWT – Food Science and Technology, 38(6), 657–663. https://doi.org/10.1016/j.lwt.2004.08.005

[fsn3658-bib-0026] Tigist, M. , Workneh, T. S. , & Woldetsadik, K. (2013). Effects of variety on the quality of tomato stored under ambient conditions. Journal of Food Science and Technology, 50(3), 477–486. https://doi.org/10.1007/s13197-011-0378-0 24425942 10.1007/s13197-011-0378-0 PMC3602550

[fsn3658-bib-0027] Urbanyi, G. , & Horti, K. (1989). Color and carotenoids content of quick‐frozen tomato cubes during frozen storage. Acta Alimentaria, 18, 247–267.

[fsn3658-bib-0028] Vinha, A. F. , Barreira, S. V. P. , Castro, A. , Costa, A. , & Oliveira, M. B. P. P. (2013). Influence of the storage conditions on the physicochemical properties, antioxidant activity and microbial flora of different tomato (*Lycopersicon esculentum L*.) cultivars. Journal of Agricultural Science, 5(2), 118–129.

